# Stabilization of Rice Bran: A Review

**DOI:** 10.3390/foods12091924

**Published:** 2023-05-08

**Authors:** Neşe Yılmaz Tuncel

**Affiliations:** Department of Food Technology, Faculty of Applied Sciences, Onsekiz Mart University, Çanakkale 17100, Turkey; neseyilmaz@comu.edu.tr; Tel.: +90-2862180018 (ext. 16097)

**Keywords:** rice bran, stabilization, by-product, utilization, sustainability, extrusion, microwave, infrared, lipase activity, free fatty acid

## Abstract

One of the major problems in food science is meeting the demand of the world’s growing population, despite environmental limitations such as climate change, water scarcity, land degradation, marine pollution, and desertification. Preventing food from going to waste and utilizing nutritive by-products as food rather than feed are easy and powerful strategies for overcoming this problem. Rice is an important staple food crop for more than half of the world’s population and substantial quantities of rice bran emerge as the main by-product of rice grain milling. Usually, rice bran is used as animal feed or discarded as waste. Although it is highly nutritious and comprises many bioactive compounds with considerable health benefits, the rapid deterioration of bran limits the exploitation of the full potential of rice bran. Hydrolytic rancidity is the main obstacle to using rice bran as food, and the enzyme inactivation process, which is termed stabilization, is the only way to prevent it. This study reviews the methods of stabilizing rice bran and other rice-milling by-products comprising rice bran in the context of the efficiency of the process upon storage. The effect of the process on the components of rice bran is also discussed.

## 1. Introduction

Scientists are searching for innovative strategies to increase food production without jeopardizing food security and biodiversity. Within the framework of sustainability, reducing food waste and utilizing by-products generated during processing are among the main objectives, considering population growth and limited resources. Rice is a major crop worldwide and rice bran (RB) is one of the most underutilized by-products of the rice milling process, despite its high nutritive, functional, and bioactive properties. 

**Figure 1:**
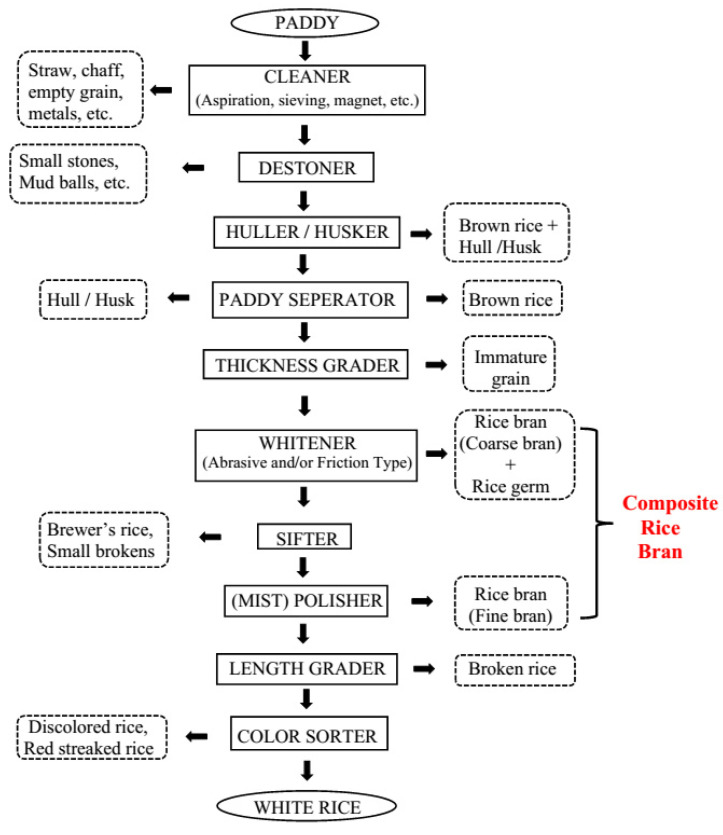
A typical multi-stage commercial rice milling system.

The hulled form of rice harvested from the field is called paddy. Milling of paddy yields around 60% white rice as the main product and approximately 10% broken rice, 20% husk, and 10% RB as the by-products [1]. The first step in rice milling is the removal of the hull or husk (Figure 1). Rice husk or hull is the coating on the seed and contains approximately 50% cellulose, 25–30% lignin, and 15–20% silica [2]. Therefore, it has no value as food. It is mostly used for energy production. Biochar from carbonized rice husk is also used as a soil amendment, activated carbon, processing fertilizer, etc. [3]. 

Brown rice, also called cargo, is unpolished whole-grain rice with the inedible rice husk or hull removed. The second step in milling involves removing bran layers to obtain white rice. Although it is known that the nutritional quality of brown rice is higher, white rice consumption is preferred all over the world. Brown rice is not well accepted mainly because of its flavor and taste. Moreover, brown rice has a coarse texture and a “dirty” look, requires longer cooking time, is more expensive, is not conveniently available, has a shorter shelf life, and gets rancid in the long term if it is not processed using a suitable stabilization process [4,5,6].

RB is the by-product produced as a result of milling brown rice into white rice. This is a multi-stage process employed by sophisticated milling systems at industrial scale (Figure 1). Multi-stage milling reduces the mechanical stress and heat buildup in the grain, thereby minimizing grain breakage and increasing head rice yield. The yield of head rice, unbroken white rice, is the basis for grading rice quality and establishing the market value. The bran layer is removed by whitening and polishing mills. Whitening machines remove the bran layers and rice germ and polishing machines remove the remaining bran by polishing the exterior of the milled kernel, which improves the appearance of milled or white rice. Composite RB consists of coarse bran fraction from the whitening steps and fine bran fraction or polish (the part of the bran fractions containing the most endosperm) from the polishing step. Composite RB may also contain rice germ and tiny fractions of rice hull (Figure 1) [7]. 

Fresh RB has great potential as a food product. It is highly nutritious and has a characteristic bland flavor and fine texture. However, unprocessed RB becomes rancid very quickly after the milling process, which limits its use as food. The rancidity of RB is predominantly triggered by lipophilic enzymes, mainly lipases. In intact paddy, lipases are primarily found in the seed coat and most of the oil is localized in the aleurone layer and rice germ. In other words, lipolytic enzymes and their substrates are physically separated in unmilled paddy rice. However, the milling process disrupts this individual localization and lipase enzymes come into contact with fat, causing hydrolysis of fat into free fatty acids (FFA) and glycerol [8]. High concentration of FFA causes a rancid soapy taste and induces oxidation and even lipotoxicity. 

RB contains several types of lipases as well as glycolipases, phospholipases, and esterases [9]. So far, two types of lipases have been purified from RB. Lipase I has a molecular mass of 40 kD and an optimum pH of about 7.5. Lipase I preferentially cleaves fatty acids from the *sn*–1 and *sn*–3 positions of triacylglycerols and is activated by calcium. On the other hand, lipase II has a molecular mass of 32 kD, a pI of 9.1, and an optimum pH of about 7.5 [10]. In addition to endogenous lipases, lipases of microbial origin can also initiate hydrolytic deterioration. Dehulling causes surface damage and disrupts the aleurone and germ (RB-oil-concentrated parts of the kernel), and lipase-producing mold and bacteria found on kernel surfaces interact with bran oil, which results in an increase in FFA [9].

The world production of rice was 787 million metric tons in 2021 [11]. Considering rice production worldwide, approximately 80 million tons of RB is produced as a by-product and is mainly utilized as feed as many food by-products. However, processed RB has great potential as a value-added commodity in the food industry. RB has been used in bakery products such as bread [12,13,14,15,16], noodles or pasta [14,17], crackers [18], biscuits [19,20], extruded snacks [21], breakfast cereals, muffins, pancakes, cookies, cakes, pies, and wafers [14,19,22], as well as a protein supplement [20,22], a binder or fat substitute in meats and sausages [22,23], ingredient in plant-based meat analogs [24], and as a beverage base [25]. RB also has great potential in applications as an ingredient in infant formulas and gluten-free products due to it being highly soluble, hypoallergenic, and gluten-free [26,27]. In addition to direct utilization, RB is also used as a source of RB protein concentrate [26,27,28,29,30,31], RB oil [32,33], RB fiber [34,35,36], RB wax [37,38], γ-oryzanol [39], and a phytochemical-rich ingredient [40]. Furthermore, RB is not only used for culinary purposes but also in the pharmaceutical and cosmetic industries. RB-derived ingredients are used in hair or skin care products, sunscreen formulations (due to natural sun protection factors), shampoos, bath oils, foundations, and various cosmetics [41].

## 2. Rice Bran Stabilization Methods 

In order to utilize RB as food instead of feed, it is essential to apply a process that will stop lipolytic activity, which is called “stabilization”. In other words, stabilization is an enzyme inactivation process that enables RB to be incorporated into the human diet. Stabilization refers to the prevention of hydrolytic degradation. Therefore, oxidative deterioration was not covered in the context of stabilization. Lipase activity and FFA formation are the main measures of hydrolytic degradation in RB. To a lesser extent, lipoxygenase and peroxidase activities were also used as RB deterioration indicators. However, FFA is the most widely used indicator due to its ease of determination. In general, RB with an excess of 10% FFA is accepted as unsuitable for human consumption [42]. An increase in FFA occurs very rapidly in freshly milled RB without proper stabilization treatment and FFA levels can reach 10% within hours depending on the post-harvest conditions. Hydrolytic rancidity development can also be avoided by rapid oil extraction soon after the rice milling process [43,44]. However, in practice, milling of rice and extraction of RB oil are not performed consecutively and the time lapse between milling and extraction results in excess amounts of FFA. However, delaying hydrolytic degradation using a stabilization process may save time for good-quality oil extraction economically from RB. 

A review of the literature on methods whose main objective is RB stabilization, clearly defined stabilization conditions, and studies of storage following stabilization is presented in Table 1. Stabilization studies carried out using chemicals, acids, or similar substances that are not edible or may be inconvenient for human and animal consumption, and studies with plant-derived extracts of unknown purity are excluded. Although they have the effect of delaying rancidity to a certain extent, studies employing physical stabilization strategies such as low-temperature storage or refrigeration were also excluded since these methods have serious limitations such as restoration of enzyme activity at room temperature and oxidative rancidity.

It was observed that extrusion, microwave (MW) heating, hot air heating, autoclaving, infrared (IR) heating, ohmic heating, radio frequency (RF) heating, ultraviolet (UV) treatment, ultrasound treatment, γ-irradiation, antioxidant addition, enzyme addition, phenolics addition, parboiling, toasting, roasting, and steaming are the RB methods for stabilization studied in the literature. 

Some of the studies on RB stabilization examined solely the effect of the stabilization process on nutritional and/or bioactive components, and did not examine the effectiveness of the stabilization itself, nor did they analyze any of the stabilization markers such as FFA or lipase activity [45,46,47,48]. 

**Table 1:** Studies on rice bran stabilization (2000–2023) following the effect of stabilization during storage.

Method and Conditions of Stabilization	Measure of Stabilization	Effect of Stabilization upon Storage	Co-Effect of Stabilization	Reference
Hot air drying at 110 °C for 2 h, Solar drying (8 h per day, max 42 °C), Steaming at 100 °C for 30 min	FFA	FFA levels of hot-air-dried, solar-dried, and steamed-RB were 9%, 39%, and 6%, respectively, while the FFA content of unstabilized RB was 78% at the end of 50 days.	Steaming showed the best results, with only 3% reduction in oil yield after 50 days in comparison with 89% oil reduction forunstabilized bran.	[49]
Extrusion at 125–130 °C for 30 s and held in the auger at 97–99 °C for 3 min	FFA	FFA levels of extruded rice bran increased from 2.8% to 3.2% and 3.3% in vacuum packs and zipper-top bags, respectively, at the end of 8 weeks when stored at 4–5 °C.	Water absorption capacity increased while fat absorption capacity decreased after the treatment compared with untreated raw bran. Emulsification and foaming capacities of extruded brans were significantly lower than that of raw bran.	[9]
Extrusion with a co-rotating twin-screw extruder (screw speed 140 rpm) at 130 °C for 20 s	Acid value	No significant change in the acid value of extruded RB for at least 20 days of storage at 25 °C.	Extrusion reduced the extractability of phytic acid.	[50]
Dry heat stabilization at 120 °C for 30 min Extrusion under the following conditions: Water flow rate: 0.000038 m^3^/s, feed rate: 27 kg/h, steam supply: 275.80 kPa, die opening: 0.0078 m, temperature: 135–140 °C. Extrudates were air dried at 50 °C for 24 h and then ground	FFA	FFA content of raw bran increased from 4.05% to 64.60%, while FFA content of dry-heated RB increased from 3.66% to 9.15%. Variation in FFA content (from 3.85% to 4.10%) of extruded bran during storage at ambient temperature in polyethylene bags for 60 days was insignificant (*p* > 0.05).	Slight increase in crude fat content, no significant change in protein content and calorific value, reducing, non-reducing, and total sugar contents, significant loss of lysine bioavailability, significant reduction in phytic acid content, significant increase in bulk density, water absorption, damaged starch content, significant decrease in fat absorption, protein solubility, and darker color were observed in stabilized brans compared with raw bran.	[51]
Extrusion (at 135 °C, 6% moisture addition, 5 s) Roasting (RB heated to 70, 90, 100, and 105 °C in a steam jacket roaster with four chambers) Pelleting (RB was conditioned with steam to increase the temperature to 90 °C, passed through a die of 5 mm, and cooled) Antioxidant addition (at 125, 250, and 375 ppm levels)	FFA Peroxide value Iodine value	Raw and pelleted RB behaved similarly in terms of storage stability. Addition of antioxidant to rice bran was not effective for stabilizing FFA, peroxide, and iodine values at any level. Extrusion was partly effective, resulting in 49.5% FFA as an average over 345 days of storage under ambient conditions.		[52]
Extrusion with pH modification (addition of HCI or Ca(OH)_2_ at the levels of 1, 5, and 10%; extrusion temperature 110–140 °C; feed moisture 20, 30, and 40%	Spectrophotometric determination of FFA and copper acetate complex	10% of HCI addition provided the lowest FFA increase at the end of 98 days of storage at room temperature (25 ± 3 °C).		[53]
Extrusion (four zones: 70 °C, 90 °C, 110 °C and 130 °C, with screw speed set at 300 rpm) Microwave heating (800 W, 75 s) Dry heating (130 °C, 120 min) Steaming (30 min) High-pressure steam (121 °C, 0.1 MPa, 15 min)	Lipase activity Lipoxygenase activity Acid value Peroxide value	After 28 days of accelerated storage at 37 °C, the acid value of untreated RB was about 6 mg KOH/100 g, while the acid values of stabilized samples were around 1.7 KOH/100 g, except for atmospheric steam-treated RB.	High-pressure steam-treated, steam-treated, and extruded RB samples were more suitable for long-term storage and better maintained the stability of flavor.	[54]
Microwave heating at 850 W for 3 min (moisture content was adjusted to 21% before the treatment)	FFA	No significant change in FFA content of MW-treated RB stored at 4–5 °C at the end of 16 weeks.	No dramatic change in proximate composition and fatty acid composition as a result of MW stabilization.	[55,56]
Microwave heating at 550 W, 2450 MHz for 3 min (moisture content was adjusted to 21% before the treatment). The temperature of the bran was 107 °C after the process.	FFA	FFA content of MW-treated RB increased from 3.2% to 3.9% in both vacuum and zipper-top packs at the end of 8 weeks when stored at 4–5 °C.	Fat absorption, emulsification, and foaming capacities of MW-treated RB were significantly lower than that of raw bran. However, MW treatment increased water absorption capacity.	[9]
Microwave heating at 700 W, 2450 MHz for 1, 3, and 5 min (moisture content was adjusted to 21% before the treatment). MW power densities were 2, 4, and 6 W/g	FFA Peroxide value	FFA content of RB treated with MW at a power density of 6 W/g for 5 min was 1.12%, while FFA of untreated control was 58.5% at the end of 28 days of storage at ambient temperature.	Protein content and oil yield significantly decreased under the noted effective MW stabilization condition.	[57]
Microwave heating Flowing MW drum heater with a max power of 6 kW (6 magnetrons × 1000 W, 2450 MHz)	Lipase activity Lipoxygenase activity FFA Peroxide value	Residual lipase activity was 13.21 U/kg for untreated control and only 1.25 U/kg for MW-treated RB at 5 kW for 13 min. Lipoxygenase activity was completely destroyed after 7 min of MW processing.	No significant decrease in fatty acid, tocopherols, and γ-oryzanol content of bran treated under optimum conditions (4 kW, 10 min).	[58]
Microwave heating at 850, 925, and 1000 W for 3, 4.5, and 6 min	FFA Acid value Peroxide value	Stabilization was investigated in 3 RB milling fractions and composite RB. Stabilization at 925 W for 3 min was recommended as the suitable condition for stabilization of RB milling fractions.	–	[59]
Infrared heating Short-wave IR radiation at 200, 300, 400, 500, 600, 700, 800, and 900 W for 1, 2, 3, 4, 5.2, 6.3, 7.2, 8.3, and 10 min	FFA	No significant increase was observed in FFA content of RB treated at IR powers of 600 W (for 4 and 5 min) and 700 W (for 2 and 3 min), while FFA content of raw RB increased from 4.32% to 43.08% at the end of 6 months at 25 °C.	The effect of IR stabilization was insignificant on γ-oryzanol content and fatty acid composition of RB. However, a significant decrease in tocopherol content of RB of up to 50% was determined.	[60]
Infrared heating Medium wave IR radiation at 500, 600, and 700 W IR power for 3.0, 5.5, and 7.0 min	FFA	Stabilization was investigated in 3 RB milling fractions. Stabilization at 700 W IR power for 7.0 min provided 90 days of shelf life without a notable change in FFA content of RB fraction obtained from the first whitening step.	Total tocopherol and γ-oryzanol contents of stabilized RB fractions were higher than in their crude counterparts.	[61]
Infrared heating (Simultaneous drying and stabilization) RB was heated for 55s to reach a surface temperature of 60 °C during each drying pass and tempered in an incubator set at 60 °C for various durations (1, 2, 3, 4, and 5 h).	FFA	It took 7 days for the FFA level of control RB to reach 10%. This period has been extended to 38 days for IR-treated RB.	IR heating at the employed conditions did not have a negative effect on milling quality.	[62]
Infrared heating with a max power of 2400 W, unspecified radiation intensity at 100, 120, and 140 °C for 5, 10, 15, and 20 min.	FFA Peroxide value	FFA content of raw RB increased from 4.4% to 62.8% after 6 months of storage at 25–30 °C, while RB treated with IR at 140 °C for 15 and 20 min maintained FFA content at around 7%.	IR stabilization at 140 °C for 15 min did not cause a significant decrease in γ-oryzanol content and fatty acid composition, but significantly decreased E vitamins.	[63]
Infrared heating with a laboratory-scale ceramic infrared drying device until the surface temperature of the rice bran reached 85 °C.	Lipase activity Lipoxygenase activity Peroxidase activity FFA Peroxide value	FFA content of untreated RB increased from 5.41% to 49.87%, and from 5.21% to 14.11% for IR-treated RB at the end of 20 days of storage at 20 °C. Lipase, lipoxygenase, and peroxidase activities in IR-treated samples were significantly higher at the end of the storage.	The contents of palmitic acid (C16:0), oleic acid (C18:1), and linoleic acid (C18:2) were well maintained by IR treatment during the whole storage period. The aroma of fresh RB was preserved.	[64]
Infrared heating either with medium or shortwave IR emitters at 700 W for 3 min	FFA	Hydrolytic lipid degradation occurred more likely in samples stored in bran form; however, samples stored in oil form were more prone to oxidative degradation. Medium-wave IR radiation was more effective in terms of retarding FFA increase compared with short-wave IR radiation.	Peroxide values of either raw or IR stabilized samples were <10 meq/kg at the end of 6 months of storage at 25 °C. Conjugated dienoic acids and *p*-anisidine values increased in all samples during storage. Storage in oil form resulted in a higher loss of total tocopherol and γ-oryzanol compared with storage in bran form.	[43]
Ohmic heating using an alternating current of 1 or 60 Hz and an electric field strength of 100 V/cm.	FFA	FFA content of RB (moisture content adjusted to 21%) stabilized with ohmic heating at 60 Hz increased from 3.25% to 5.47%, while FFA content of raw RB increased from 3.96% to 18.03% when stored in Ziploc bags at 4 °C for 6 weeks. The moisture content of the sample had a very decisive effect.	Increase in rice bran oil extraction yield.	[65]
Ohmic heating Using an alternating current of 50 Hz and an electric field strength of 75, 150, and 225 V.cm^–1^. Moisture content of RB was adjusted to 20, 30, and 40%.	FFA Lipase activity	Lower FFA levels and lipase activity were observed in ohmically treated RB compared with raw RB stored at 4 °C for 21 days. The moisture content of the sample had a very decisive effect.	A very slight increase was observed in total phenolic, α-tocopherol, and γ-oryzanol levels of ohmic-heated RB compared with raw RB.	[66]
Ohmic heating at 20, 30, and 40% initial moisture content, 132, 150, 168, 189, 216 V, and 50 Hz. Electrical field strengths were 44, 50, 56, 63, and 72 V/cm.	FFA Peroxide value	FFA of ohmically heated bran was 4.77% after 75 days of storage, whereas it was 41.84% for raw RB.	The peroxide value of ohmically heated samples after 75 days of storage was 4.7 meq/kg.	[67]
Hot air-assisted radio frequency (HA-RF) heating using a 6 kW, 27.12 MHz pilot-scale free-running oscillator RF system combined with a hot air oven	FFA Peroxide value Lipase activity Lipoxygenase activity	RB treated by HA-RF heating to 100 °C with 15 min holding can be stored at 35 °C and remain below acceptable thresholds for a period of 60 days without adverse effects on product quality.	Optimum HA-RF heating treatment led to a significant increase in tocopherol content, but had no significant effect on γ-oryzanol.	[68]
Radiofrequency heating at 5 kW, 40.68 MHz for 2 min	FFA Acid value Peroxide value Lipase activity	Lipase activity retention was close to zero after 2 min of RF heating. FFA content of untreated bran stored at 37 °C was almost 4-fold higher than that of RF-treated bran after 8 weeks. Peroxide values of either raw or processed brans were below 10 meq O_2_/kg for 8 weeks and at all storage temperatures (4, 25, 37 °C).	No significant difference in total phenolic and flavonoid contents, DPPH scavenging activity, reducing power, and color between the untreated control and radio frequency-treated RB (*p* > 0.05)	[69]

### 2.1. Extrusion

Extrusion is one of the oldest and possibly the most widespread method of RB stabilization. Generally, temperatures between 120 and 130 °C were sufficient to inactivate RB lipase. Randall et al. (1985) reported that no notable increase was observed in FFA content of RB extruded at 130 °C during storage at 32 °C and 85% relative humidity (RH) for 28 days [70]. The authors stated that although a consistent temperature of 120 °C was usually suitable, stabilization was always sufficient at 130 °C. However, FFA content of raw bran that was stored at 32 °C increased to over 80% [70]. Additionally, Kim et al. (1987) reported a complete inactivation in lipase activity at temperatures above 128 °C regardless of the moisture content of the RB fed to a single screw extruder [71]. Similarly, Fuh and Chiang (2001) indicated that extrusion at 130 °C for 20 s with a screw speed of 140 rpm was sufficient to inactivate lipase [50]. Shin et al. (1997) extruded RB at 110, 120, 130, and 140 °C with post-extrusion times of 0, 3, and 6 min and stored RB at ambient temperature for 375 days [72]. FFA content of raw RB reached over 70% at the end of the year. Although the FFA level of the processed RB was below 7% at all extrusion temperatures after 1 year, it was observed that the amount of FFA content increased as the extrusion temperature decreased during storage. In addition, it was reported that post-extrusion holding time had no effect on FFA levels in extruded RB [72]. 

Malekian et al. (2000) carried out extrusion (125–130 °C for 30 s) with the aim of RB stabilization. FFA levels of raw (untreated) and extruded RB samples were 26.7% and 3.2% when stored in vacuum packs, and 22.2% and 3.3% when stored in zipper-top packs, respectively, at the end of 8 weeks of storage at 4–5 °C [9]. It was observed that for untreated RB, vacuum-packed samples had a higher increase in FFA levels compared with samples in zipper-top bags. The authors attributed this result to anaerobic microorganisms present in RB [9]. Sharma et al. (2004) stabilized RB using dry heating (120 °C for 30 min) and extrusion cooking (135–140 °C) [51]. The authors stated that they observed an incomplete destruction of lipase in dry heat-treated RB since FFA content increased from 3.66% to 9.15% at the end of 60 days of storage at ambient temperature. However, extruded RB showed no significant increase during storage under the noted conditions [51]. Escamillo–Castillo et al. (2005) also studied extrusion stabilization of RB with pH modification [53]. The authors added HCI or Ca(OH)_2_ to the RB samples at the levels of 1, 5, and 10% before extrusion. Although extrusion alone or in combination with any of the chemicals resulted in lower FFA when compared with the unprocessed RB, the authors concluded that the addition of Ca(OH)_2_ promoted the activity of lipases and led to higher FFA concentrations during storage. The lowest FFA increase was observed in RB samples treated with 10% HCI, regardless of the initial moisture content of the bran [53]. Rafe and Sadeghian (2017) extruded RB at temperatures between 100 and 130 °C and the lowest lipase and peroxidase activities were obtained at 123 °C die temperature, 354 rpm screw speed, and 10.8% initial moisture content [73]. 

### 2.2. Microwave Heating

Many researchers proposed MW heating as an efficient RB stabilization process [42,55,56,57,58,59,74,75]. However, it should be noted that household MW ovens were used in almost all these studies. Although attempts to increase homogeneity are made by rotating the sample, it is well recognized that domestic scale MW ovens can suffer from non-uniform heating, resulting in cold and hot spots. However, industrial scale MW ovens may provide more uniform heating since they can have different designs, magnetron placements, modes, and dimensions of cavity. 

Tao et al. (1993) stabilized RB with MW heating at 2450 MHz for 3 min and found that the FFA content of MW-treated long grain RB increased from 4.0% to 4.9% and that of medium grain RB increased from 4.6% to 6.25% at the end of 4 weeks of storage at 33 °C and 75% RH. On the other hand, FFA content of untreated raw RB ranged from 4.6% to 56.8% and 4.0% to 68.3% in medium and long-grain bran, respectively [42]. Ramezanzadeh et al. (1999) adjusted the moisture content of RB (150 g per batch) to 21% and heated the moistened RB in plastic zipper-top bags at 850 W for 3 min in a household MW oven [55]. The temperature of the heated RB was 107 ± 2 °C after the process. The FFA content of raw RB increased from 2.5% to 54.9% and 48.1% at 25 °C to 25.4% and 19.5% at 4–5 °C at the end of 16 weeks of storage in vacuum bags and zipper-top bags, respectively. However, FFA content of MW-heated RB increased from 2.8% to 6.9% and 5.2% at the end of the storage period when stored in vacuum and zipper-top bags, respectively, at 25 °C, while no significant change was observed in FFA content of MW-heated RB when stored at 4–5 °C [55]. 

Patil et al. (2016) reported that the FFA content of RB treated with MW at a power density of 6 W/g for 5 min was 1.12%, while the FFA content of untreated control was 58.5% at the end of 28 days of storage at ambient temperature [57]. Ertürk and Meral (2019) compared MW and conventional heating with regard to RB stabilization [75]. Although the researchers did not conduct a storage study, it was reported that lipase activity was significantly decreased by both processing methods in proportion to MW power or oven temperature, although MW treatment had a greater effect [75]. Li et al. (2018) stabilized RB using a flowing MW drum heater under different conditions of MW power, duration time, and ventilation rate [58]. FFA contents of RB samples treated with MW at 4 and 5 kW for 10 min were below 3% at the end of 60 days of storage at 35 °C. Significant and negative correlations were reported either between lipase activity and final bran temperature at the end of the process or between lipase activity and process time. Researchers stated that LOX activity was completely destroyed after 7 min of the MW process, even at 3 kW, and residual lipase activity was only 1.25 U/kg in RB treated with MW at 5 kW for 13 min, while it was 13.21 U/kg for the untreated control [58]. Process durations were notably longer than those reported in RB stabilization studies carried out using household MW ovens and it was clearly shown that longer process time is more effective for stabilization. Therefore, it can be interpreted that even with the same method, different results may be obtained in studies where the processing equipment is different. 

### 2.3. Dry or Moist Heat Treatments

Stabilization of RB using dry or moist heat treatments such as hot air heating, toasting, roasting, steaming, and autoclaving is also one of the most widely employed techniques. Thanonkaew et al. (2012) reported that hot air heating at 150 °C for 10 min, roasting at 150 °C for 2 min, and steaming at 130 °C for 2 min with domestic kitchen equipments resulted in lower FFA, acid value, and peroxide value compared with raw RB [76]. However, the researchers did not perform any storage study and measured the noted parameters only after the process [76]. Amarashinge et al. (2009) reported that FFA contents of unstabilized, solar-dried (8 h per day, max temperature 42 °C), hot-air-dried (110 °C, 2 h), and steamed (100 °C, 30 min) RB were 78%, 39%, 9%, and 6%, respectively, at the end of 50 days of storage [49]. The researchers concluded that steaming showed the best results, with only 3% reduction in oil yield and the lowest FFA content after 50 days. Many studies have reported that moist heating treatments (i.e., steaming or pre-moisturization) provide a more effective stabilization than dry heating [49,77,78]. Brunschwiler et al. (2013) showed that heating RB with a moisture content of 20% at 110 °C for 5 min almost completely inactivated lipase/esterase activity (0.3% of the activity in raw RB). However, the treatment conditions required to achieve the same inactivation rate in RB with 10% moisture content was heating at 120 °C for nearly 40 min [78]. 

Li et al. (2023) analyzed the efficiency of different stabilization treatments and their effects on the flavor of RB based on GC–MS, E-nose, and E-tongue analyses [54]. The researchers reported that MW and high-pressure steam treatments were effective in terms of retarding FFA increase and passivating lipase activity, while atmospheric steaming had the worst effect. The content of total volatile compounds in extruded and MW-treated RB was lower than that of untreated RB, whereas untreated and steam-treated RB had almost the same content of total volatile compounds. Bitterness increased slightly during storage. It was concluded that flavors of high-pressure steam-treated, steam-treated, and extruded RB were similar and can better maintain the stability of flavor, which makes these stabilization methods more suitable for long term RB storage [54]. 

### 2.4. Infrared Heating 

The first study on infrared stabilization of RB was reported by Yılmaz et al. (2014) [60]. The researchers found no significant increase in the FFA content of RB treated with IR radiation (using short-wave IR emitters) at 600 W IR power for 4 and 5 min and 700 W IR power for 2 and 3 min at 25 °C over 6 months, while the FFA content of raw RB increased from 4.32% to 43.08% at the end of storage [60]. Irakli et al. (2018), who also employed IR radiation (unspecified radiation intensity), reported very similar results [63]. It was found that the FFA content of RB treated with IR radiation at 140 °C for 15 and 20 min was around 6–7%, while FFA concentration in raw RB increased from 4.4% to 62.8% during 6 months of storage at 25–30 °C [63]. 

Yan et al. (2020) used a laboratory-scale ceramic IR drying device to stabilize RB [64]. The researchers employed IR heating until the temperature of the bran surface reached 85 °C; however, they did not justify why they chose this temperature. It was found that lipase activity of the IR-treated RB was lower than that of the untreated RB. Nonetheless, lipase activity of the IR-treated bran also significantly increased during the storage period (20 days at 20 °C). Although they showed a fluctuating trend, both lipoxygenase and peroxidase activities of the IR-treated RB significantly increased during the storage period. Additionally, FFA content of the IR-treated samples significantly increased during storage, albeit with a lower acceleration compared with control samples [64]. Wang et al. (2017) carried out a simultaneous rough rice drying and RB stabilization study using IR radiation [62]. The authors used a catalytic IR emitter and reached a rice surface temperature of 60 °C and then tempered the samples in an incubator at 60 °C for different durations ranging from 1 to 5 h. Although FFA levels increased during the course of storage (38 days at a temperature of 20 ± 1 °C and RH of 46 ± 3%) under all tested conditions, it was reported that the time until the FFA reached 10% was extended to 38 days compared with 7 days for the untreated control sample [62]. 

It should be noted that the intensity and penetration power of IR radiation is strongly dependent on the IR emitter used. Yılmaz Tuncel and Yılmaz Korkmaz (2021) reported that medium-wave IR heating was more effective at retarding the increase in FFA compared with short-wave IR heating when employed at the same IR emitter power and process time (700 W, 3 min) [43]. It was found that the FFA content of short-wave IR-stabilized samples was significantly higher than that of medium-wave IR-stabilized counterparts during 6 months of storage at ambient temperature [43]. Similar findings were also reported by Atungulu and Pan (2011) who indicated that foodstuffs absorb medium-wave IR energy more efficiently; however, absorptivity decreases and transmissivity increases in short-wave IR processes, especially for thin materials [79].

### 2.5. Ohmic Heating 

Ohmic heating generates heat through passage of electrical current through food which resists the flow of electricity and can be used in enzyme inactivation [80]. Lakkakula et al. (2004) used ohmic heating to stabilize RB and to improve the yield of RB oil [65]. It was noted that FFA contents of raw and electrically heated RB were 18.0% and 5.5%, respectively, at the end of 6 weeks when stored at 4 °C. Researchers reported that although the process had a limited effect on FFA levels—probably due to the electroporation phenomenon—ohmically heating RB without moisture adjustment (adding water) was not effective due to the low electrical conductivity of the bran, which has an intial moisture content of around 10% [65]. 

Stabilization of RB with ohmic heating was also studied by Loypimai et al. (2009) [66]. The authors used an alternating current of 50 Hz and applied 75, 150, and 225 V.cm^–1^ electrical field strengths to RB with moisture contents of 20, 30, and 40%. Although it was shown that ohmically heated RB had a lower FFA level and lipase activity, the effect of different ohmic processing conditions on these parameters was not so clear since the authors stored samples at 4 °C for only 21 days. However, it was shown that lipase activity of ohmically treated samples with a moisture content of 20% was higher than that of samples with 30% and 40% moisture content [66]. Dhingra et al. (2012) also stated that the FFA content of ohmically heated RB was 4.77% after 75 days of storage, whereas it was 41.84% for raw bran [67]. However, the authors did not indicate the conditions of storage. In all the aforementioned studies, it was observed that the moisture content of the sample had a critical effect on the efficiency of the process. 

### 2.6. Radio Frequency Heating 

Ling et al. (2018) used hot air-assisted RF heating (6 kW, 27.12 MHz) combined with a hot air oven to stabilize RB [68]. In this study, significant decreases in residual lipase and lipoxygenase activities after treatment under specific conditions were observed; however, it was found that the activities of the noted enzymes increased again during storage, probably due to water absorption. The researchers concluded that stabilization methods using dry heating may not be successful at irreversibly inactivating lipase and lipoxygenase activities, especially when the moisture content of the bran increased to atmospheric equilibrium during storage. Nevertheless, hot air-assisted RF heating at 90 °C for 30 min resulted in 4.01% of FFA, while the FFA content of the untreated control sample was 50.67% at the end of 60 days of storage at 35 °C and 70% RH [68].

Chen et al. (2021) also employed RF heating (5 kW, 40.68 MHz) to stabilize RB and reported that lipase activity retention was close to zero after 2 min [69]. However, the increase in FFA content during storage could also not be prevented, even at 4 °C [69]. Liao et al. (2020) did not perform FFA analysis or storage study; however, they showed that relative lipase, polyphenol oxidase, and peroxidase activities of RB treated with high-temperature hot air-assisted radio frequency (110–115 °C, 6 min) were 20.1%, 22.9%, and 7.6% that of raw bran, respectively [81]. 

### 2.7. Irradiation

Shin and Godber (1996) irradiated RB at 5, 10, and 15 kGy doses using a Cobalt-60 source [82]. The authors found higher levels of FFA in irradiated RB compared with untreated bran after the process, and FFA levels reached almost 90% at the end of 52 weeks of storage at ambient temperature (22–26 °C). It was concluded that γ-irradiation of RB did not decrease lipolytic enzyme activity in the range used. Significant losses were also reported for E vitamins and γ-oryzanol in irradiated RB [82]. However, positive effects of γ-irradiation have also been reported in other studies whose aim was not stabilization. For instance, Masamran et al. (2023) applied γ-irradiation to defatted RB before protein extraction and reported that the extraction yield and protein recovery increased with the treatment. The authors also concluded that γ-irradiation changed the structure of RB and increased the release of bioactive compounds such as phenolic compounds and resulted in enhanced antioxidant activity in irradiated extracts [83]. 

### 2.8. Other Stabilization Approaches 

Pourali et al. (2009) employed subcritical water extraction as an environmentally friendly technique to stabilize and extract RB oil simultaneously [84]. Researchers also conducted conventional solid–liquid extraction (hexane–bran, hexane–water–bran, and ethanol–bran) for comparison. No increase was observed in the content of FFA in subcritical-water-extracted and ethanol-extracted (60 °C) RB oil, while FFA concentration increased from 5.0% to 5.6% and from 6.5% to 7.0% in hexane-extracted (25 °C) and hexane–water-extracted (25 °C) RB oils, respectively, after 12 weeks of storage [84]. Another interesting approach was performed by Raghavendra et al. (2017) [85]. It is known that polyphenols can bind proteins and enzymes and alter their structural properties and biological activities. Raghavendra et al. (2017) showed that the activity of isolated and purified RB lipase decreased in the presence of chlorogenic and prominently caffeic acids. Researchers found 56% loss of lipase activity at 60 µM caffeic acid concentration. The loss of enzymatic activity increased with increasing concentration of the noted ligands [85]. 

Gopinger et al. (2015) treated RB with a mixture of acetic and propionic acids (1:1, m/m) with the aim of stabilization. The organic acid mixture (2% based on bran weight) was applied via spraying and the bran samples were stored at +18 °C for 120 days. Although the authors did not analyze typical stabilization indicators such as lipase activity and FFA content, lower lipid acidity (titratable acidity) increase and less lipid oxidation product formation were reported in organic acid-treated RB [86]. Yu et al. (2020) compared various stabilization methods such as MW (700 W for 2, 4, 6 min), steam heating (for 20, 40, 60 min), dry heating (at 105 °C for 30, 60, 90 min), IR heating (at 105 °C for 30, 60, 90 min), autoclaving (at 121 °C for 20 min), extrusion (at 60, 65, 115, 120 °C subsequent heating, 400–500 rpm screw speed), enzyme treatment (pepsin and papain), low-temperature storage (at 4, −18, and −80 °C for 72 h), ultraviolet irradiation (at 254 nm for 6, 12, 18 h), and ultrasound (28 kHz and 300 W for 30, 60, 90 min) for acid value, lipase, and peroxidase activities [87]. The authors reported that autoclaving is the most effective method for lipase inactivation at the noted conditions. Significant decreases in acid values were found after MW, autoclaving, steam heating, low-temperature storage, IR heating, and extrusion treatments (*p* < 0.05). However, non-thermal methods were not effective in terms of lipase inactivation. Residual lipase activities of RB treated with UV radiation even for 18 h and RB stored at extreme low temperature (−80 °C for 72 h) were 57% and 58%, respectively. Moreover, ultrasound markedly increased the peroxide value of RB oil [87]. 

Parboiling is another practice employed for RB stabilization. Although the term “parboiling” does not define a specific condition, it generally refers to soaking the paddy in water (at varying temperatures) followed by a short steaming procedure and drying (solar drying in most cases). In general, the bran obtained from milling of parboiled paddy is used for extraction of RB oil [49].

## 3. Stabilization of Individual Rice Bran Fractions

In sophisticated multi-brake systems, brown rice is whitened in several stages in order to avoid heat generation, which causes higher rates of cracking and broken grains. Therefore, RB is obtained step-by-step and all of these fractions are accumulated as composite RB at the end of the process. These fractions do not directly represent the outer, middle, and inner bran layers of the grain, since RB is not removed layer-by-layer, but rather, more like scraped away piece-by-piece. Nevertheless, it is not incorrect to indicate that these fractions contain more pieces from the outermost to innermost layers of the bran as the milling progresses.

Yılmaz (2016) showed that the FFA content of the unprocessed bran fraction obtained from the last whitening machine and water-mist polisher (BF3), which contain mostly the innermost bran layer, was significantly higher than that of the unprocessed bran fractions obtained from the first (BF1) and second (BF2) whitening machines, which contain mostly the outer layers of the bran (*p* < 0.05) [61]. This result was attributed to the water-mist polishing which may have favored the lipolytic enzyme activity due to the addition of a small amount of water and an increase in temperature due to the friction forces during this milling step. Moreover, it was found that the lowest increase in the FFA content (from 4.88% to 6.00%) was observed in IR-treated (at 700 W for 3 min) BF1 during 3 months of storage at ambient temperature [61]. Similarly, Lavanya et al. (2019) found that the rancidity levels were significantly different in bran fractions, and FFA, acid value, and peroxide values significantly increased from the first fraction to the third fraction (from outer to inner RB layers) [59]. This trend continued throughout the storage of MW-stabilized fractions as well. The increase in FFA was also greater in the BF3 sample compared with the BF1 sample, which was exposed to the same MW power for the same process time [59]. Yu et al. (2022) classified RB into four fractions using a specific surface abrasion apparatus and indicated that fractions one and two mainly consisted of pericarp, testa, and nucellus, while fractions three and four mainly consisted of aleurone layer. Controversially, the researchers found that lipase activity decreased significantly from the outer fractions to the inner fractions [88].

## 4. Stabilization of Other Rice Milling by–Products Comprising Rice Bran

Brown rice or cargo is a whole grain and can be defined as the unpolished version of the rice grain consisting of RB, rice germ, and endosperm. Immature rice is also a whole grain and a form of brown rice. Immature rice is defined as either chalky, thin kernels or kernels that have green seed coats. Immature rice is produced as a by-product during the rice milling process in around 5% of the paddy milled [89]. Like the RB itself, brown rice and immature rice are also unstable in nature due to the presence of RB and require a stabilization process for long-term storage. 

A very recent study investigated the effect of cold plasma, an innovative non-thermal processing technology, on the storage stability of brown rice [90]. Researchers found that peroxidase activity was completely inactivated when cold plasma (at 160 kV for 15 min) was applied to brown rice in the form of brown rice powder. However, 61% of residual peroxidase activity was found after treatment under the same conditions when cold plasma was applied to whole brown rice grain [90]. In other words, cold plasma had a very limited effect on peroxidase activity when applied to the grain itself instead of the powder form, probably due to the low depth of penetration.

Qian et al. (2014) applied pulsed electric field (PEF) treatment to monolayer brown rice grains and reported that lipase activity could be significantly inactivated by PEF [91]. The authors found that the voltage was the most important parameter for the inactivation efficiency of the process, followed by frequency and pulse width [91]. 

Bergonio et al. (2016) investigated the effect of dry heating (at 60 °C for 15, 20, and 25 min), MW heating (800 W, 2450 MHz, for 30, 60, and 90 s), and steam (for 30, 60, and 90 s) on lipase activity and FFA content of brown rice and found that all treated brown rice samples showed significantly lower FFA content and lipase activity after the process. However, a steady and significant increase in FFA content of each treated and untreated brown rice sample was noted during storage, while the increase was significantly lower for treated samples [92]. 

Yılmaz et al. (2018) stabilized immature rice grain using either medium or short-wave IR radiation at 1000–1600 W for various process times and showed that the FFA content of untreated immature rice increased from 5.59% to 35.71% at the end of 3 months of storage at room temperature, while the increase in FFA was statistically insignificant in the samples treated with both medium and short-wave IR radiation at 1600 W for longer than 4 min [89]. 

## 5. Factors Affecting Hydrolytic Stability of Unprocessed Rice Bran

Hydrolytic stability of raw RB is affected by various factors, including variety, milling conditions, storage conditions, and the analytical procedure employed for the stability markers. 

Rice variety remarkably affects the hydrolytic stability of RB and therefore, it is hard to propose a single condition for effective stabilization. Different rice varieties milled within the same milling system under the same conditions and stored at the same temperature and RH may have different levels of FFA [43]. Considering the remarkable effect of variety, the use of breeding techniques is reported to have a potential to increase RB stability against lipid hydrolysis [93,94]. Goffman and Bergman (2003a) stated that hydrolytic rancidity was highly and significantly correlated with esterase activity but not with RB oil concentration [93]. The authors reported about two-fold higher hydrolytic rancidity and esterase activity in RB samples with low oil content (16–20%) compared with samples with high oil content (24–28%). Researchers found 58% higher FFA content in the Cypress variety, which had 26% higher lipase activity than the Earl variety at the end of 5 months of storage at room temperature [94]. It was concluded that the intensity of the hydrolytic rancidity is primarily related to lipase activity of RB and, therefore, selection of varieties with low lipase activity could be useful for increasing RB stability. Rattanathanan et al. (2022) investigated the hydrolytic stability of either regular non-pigmented RB or black RB and reported similar lipase activities (269 and 241 U/kg, respectively) [95]. Controversially, Goffman and Bergman (2003a) reported that red RB had lower values for both hydrolytic rancidity and esterase activity and attributed this result to the high tannin content that may inhibit the lipase activity [93]. 

Higher rates of FFA accumulation in the first stages of storage were reported in many studies [43,55,60]. Goffman and Bergman (2003b) indicated that depletion of RB triacylglycerols is concentration-dependent. The lipolytic activity is maximal at the beginning of storage when the triacylglycerol content is highest and decreases gradually with decreasing concentration [94]. Additionally, it was clearly shown that the food matrix is considerably decisive with regard to RB oil degradation mechanism [43]. Almost 4-fold higher FFA occurred in raw RB when stored in bran matrix (68.27%) compared with oil matrix (17.94%) at the end of 6 months of storage at 25 °C. This result was attributed to the fact that lipase exhibited enzyme activity when the substrate oil is dispersed in a medium containing considerable amounts of water [43]. Similarly, Pourali et al. (2009) showed that the FFA content of stored (at 25 °C) raw RB increased from 5.6% to 36.0%, while the FFA content of RBO extracted using hexane increased from 5.0% to 5.6% at the end of 12 weeks [84]. 

Determination of FFA content using different analytical procedures is another factor that complicates the comparison of hydrolytic stability of RB. For instance, Escamilla–Castillo et al. reported 5.25% increase in FFA (from 0.14% to 5.39%) at the end of 98 days (spectrophotometric determination), while Mujahid et al. found 87.3% increase in FFA (from 9.5% to 96.8%) after 345 days (titrimetric determination) in raw RB stored at room temperature [52,53]. 

Degree of milling, which defines the amount of bran removed from brown rice, affects the composition of the bran since it affects the bran layers or fractions included in the composite RB. Thus, the hydrolytic stabilities of RB obtained from rice grains that were milled to different degrees may be different. Moreover, some studies obtained the RB sample using laboratory-scale portable rice debranners that have completely different milling dynamics and therefore, it should be noted that studies using this type of sampling may not reflect the behavior of the commercial RB sample. 

## 6. Nutritive Value of Rice Bran and the Effect of Stabilization

RB contains significant amounts of macronutrients such as proteins, fats, and fiber and bioactive micronutrients such as vitamins, especially B group vitamins and tocols, minerals, γ-oryzanol, phytosterols, and squalene [96]. The composition of RB is presented in Table 2. Although the amount of nutrients can vary depending on factors such as rice variety, climatic conditions, agricultural practices, rice milling system, maturity level of the grain, method of analysis, type of solvents used, etc., Table 2 presents an average range of values in the literature. Stabilization methods also affect the nutritional attributes of RB, as discussed in a recent review [97]. 

Crude protein content of RB ranged between 11% and 16% [50,51,53,68,73,98,99]. The potential of RB as a protein source should be emphasized since plant-based protein sources are one of the largest growing product groups in the food market. Studies on RB protein concentrates and their properties have dramatically increased in recent years. RB protein has high nutritional value and is efficiently digested; it has a protein efficiency ratio of 1.6 [1]. Although there are discrepancies in the amino acid composition of RB protein in the literature, probably due to the variation in rice cultivars, all essential amino acids are present in varying proportions in RB protein [22,40]. It has been reported that the concentrates of RB protein have an efficiency ratio of 2.0–2.2, which is comparable to milk protein casein (2.5) [1,22]. Prakash and Ramaswamy (1996) found that threonine and isoleucine are limiting amino acids in RB protein concentrates [22]. On the other hand, Wang et al. (2015) reported that the contents of essential amino acids such as phenylalanine, valine, methionine, leucine, and histidine were higher in RB protein concentrates compared with soy protein isolates [100]. Moreover, antioxidant, anti-inflammatory, antimicrobial, antihypertensive, antidiabetic, and anticancer activities were reported for hydrolysates and peptides derived from RB protein [27]. 

**Table 2:** Nutritional composition of rice bran.

Component	Average Range	Reference
Crude fat (%)	18–23	[50,68,73,98,99,101]
Crude protein (%)	11–16	[50,51,53,68,73,98,99]
Ash (%)	8–12	[50,68,73,98,99]
Soluble dietary fiber (%)	2–5	[50,73,98,99]
Insoluble dietary fiber (%)	20–27	
Total dietary fiber (%)	22–32	
γ-oryzanol (g/kg)	0.5–5.5	[46,50,60,82,95]
Vitamins		
Total Tocopherols (mg/kg)	100–150	[60,82,101]
α-T (mg/kg)	50–130	
β-T (mg/kg)	2–10	
γ-T (mg/kg)	10–50	
δ-T (mg/kg)	0–2	
Total Tocotrienols (mg/kg)	130–170	[82,101]
α-T3 (mg/kg)	38	
β-T3 (mg/kg)	–	
γ-T3 (mg/kg)	120–140	
δ-T3 (mg/kg)	0–10	
Vitamin B1 (Thiamin) (mg/kg)	12–40	[5,50,99,102]
Vitamin B2 (Riboflavin) (mg/kg)	1–4	[5,50,99,102]
Vitamin B3 (Niacin) (mg/kg)	300–800	[5,50,99,102]
Vitamin B5 (Pantothenic acid) (mg/kg)	74	[102]
Vitamin B6 (mg/kg) (Pyridoxamine, pyridoxal, pyridoxine)	20–40	[99,102]
Minerals		
Ca (mg/kg)	300–1200	[5,16]
K (mg/kg)	5992	[16]
Fe (mg/kg)	86–430	[5,16]
Zn (mg/kg)	50–250	[5,16]
P (mg/kg)	6278	[16]

Although the crude fat content of RB has been found to be in the range of 18–23% in many studies [50,68,73,99,101], very low values such as 9% [53] and very high values such as 30% [1] have also been reported. Goffman et al. (2003) investigated the lipid content and fatty acid profiles of RB obtained from 204 genetically diverse rice accessions and found that genotype effects were highly statistically significant (*p* < 0.001) [103]. It was stated that the effect of season (year) on oil content and fatty acids was also significant, except for palmitic acid. In general, saturated and unsaturated fatty acid contents of RB oil ranged between 15 and 30% and 70 and 85%, respectively. Palmitic acid is the most abundant saturated fatty acid, while oleic and linoleic acids are the dominant fatty acids among unsaturated fatty acids [1,60,82,104]. Similar fatty acid distributions were reported for RB oil in the literature. Besides, it was reported that stabilization processes such as IR and MW did not significantly alter the fatty acid composition of RB [56,60].

RB oil contains over 4% unsaponifiable matter mainly consisting of plant sterols (mostly campesterol, stigmasterol, and β–sitosterol), triterpene alcohols (24–methylene cycloartenol and cycloartenol), tocopherols, and tocotrienols [105]. Among them, γ-oryzanol, which is a mixture of ferulic acid esters of triterpenoid alcohols, composes 20–30% of the unsaponifiable matter and 1–3% of RB oil and is one of the most important bioactive components of RB oil [1]. Gamma-oryzanol has great potential in nutraceutical, pharmaceutical, and cosmeceutical applications due to its numerous health benefits. It has been reported that γ-oryzanol has notable antioxidant (at least four times higher than tocopherols), anti–inflammatory, anticancer, and cholesterol-lowering properties. In addition, it helps to reduce platelet aggregation, fight menopausal distress, increase bile acid excretion, inhibit and tumor growth, and also has protective effects against UV light [105,106,107,108,109,110,111]. Furthermore, γ-oryzanol is a very stable compound. It has been demonstrated in many studies that there is no decrease in the amount of γ-oryzanol as a result of various stabilization processes such as IR heating [60,61,63,87], MW heating [46,58,87], ohmic heating [66], hot air heating [76] RF heating [68], autoclaving, extrusion, steam heating, ultrasound, and UV radiation [87].

Tocopherols are another important bioactive component found in RB oil. Among the tocopherol analogues, α-tocopherol is the most abundant analogue in RB oil, followed by γ-tocopherol [46,58,60,61,82,112]. Unlike γ-oryzanol, tocopherols are more susceptible to heat and showed significant decreases as a result of stabilization processes such as extrusion [72], IR [60,113], MW [58,113], and steaming [95]. 

The contents of both γ-oryzanol and tocopherols are affected by both variety and environmental factors. However, Bergman and Xu (2003) showed that growing environment has a greater effect on γ-oryzanol and tocopherol levels than genotype [112]. Many researchers found that the γ-oryzanol content of RB is many times higher than tocopherols [60,61,112]. Moreover, individual RB fractions have different levels of γ-oryzanol and tocopherols [61,114]. Yılmaz (2016) reported that the fraction obtained from the first mill, which contains mostly the outermost bran layer, is the richest in γ-oryzanol and this amount decreases towards the inner layers. However, tocopherols show the opposite trend [61]. Similarly, Britz et al. (2007) stated that γ-oryzanol is concentrated in the outermost pericarp and seed coat, while tocols are well presented in the deeper aleurone [114]. Furthermore, RB oil is the only readily available oil that contains significant levels of tocotrienols, with the exception of palm oil [41].

In addition, RB is a notable source of B group vitamins, especially niacin. Yılmaz and Tuncel (2015) found 36.92 mg/kg of thiamin (B_1_), 0.91 mg/kg of riboflavin (B_2_), 338.50 mg/kg of niacin (B_3_), 8.46 mg/kg of pyridoxamine, 1.90 mg/kg of pyridoxal, and 12.86 mg/kg of pyridoxine (23.23 mg/kg Vitamin B_6_ in total) in raw RB [99]. The authors observed a significant decrease of up to 42% in thiamin content, while no changes in the contents of riboflavin, niacin, and total B6 vitamin were observed as a result of IR stabilization [99]. Moreover, Rafe and Sadeghian (2017) reported that the content of vitamins B_2_, B_3_, B_5_, and folic acid remained unchanged as a result of extrusion [73]. 

Other important micronutrients in RB are phospholipids or lecithin (one of the major classes of lipid in RB oil), phytosterols, squalene, and phenolic acids. The main phytosterols are β–sitosterol (c. 50%), campesterol (c. 20%), stigmasterol (c. 15%), and isofucosterol (c. 5%) [115]. Phytosterols are known for their cholesterol-lowering properties due to the inhibition of cholesterol absorption in the small intestines. Squalene, which is a triterpene and intermediate metabolite of cholesterol synthesis, is found in shark fish liver oil and vegetable sources such as olive oil, palm oil, wheat-germ oil, and amaranth oil. RB oil has higher squalene content compared with commercial vegetable squalene derived from olive oil [115]. Pokkanta et al. (2022) found 1252 mg/kg of total phytosterols (stigmasterol, campesterol, β-stigmasterol), 99.55 mg/kg of squalene, 3 mg/kg of cholecalciferol, and 2.45 mg/kg of phylloquinone in RB [46]. 

RB is also a notable source of phenolics, especially in bound form. Ferulic (1863 μg/g) and *p*-coumaric (647 μg/g) acids are the most abundant phenolic acids in RB [99]. The presence of gallic acid, protocatechuic acid, 4-hydroxybenzoic acid, catechin, vanillic acid, chlorogenic acid, caffeic acid, kaempferol, epigallocatechin, trans–*p*–coumaric acid, syringic acid, and sinapic acid was also reported [46,99,116]. In general, pigmented RB extracts have higher contents of phenolic compounds and antioxidant activity [117]. 

RB contains about 50% carbohydrates, mainly starch [118]. Total dietary fiber (TDF) content of RB ranges from 22 to 32%, with less than 5–6% as soluble dietary fiber (SDF) [50,73,98,99]. Sharma et al. (2004) reported 10–20% starch, 3–8% reducing sugars, 8–11% hemicelluloses, and 10–12% celluloses in RB [51]. Parboiling may also notably affect the properties of starch in rice grain. 

Antinutrients in RB are not widely studied. The phytic acid content of raw RB was 23–93 mg/g, depending on rice cultivar, and significant decreases in phytate content were reported following extrusion [50,73,102,119,120] and IR stabilization [94]. Kaur et al. (2015) reported 53.82 TIU/g of trypsin inhibitor activity in raw RB and found a significant decrease following extrusion treatment [102].

## 7. Conclusions

In conclusion, RB is a highly nutritious and technologically functional by-product with remarkable health-promoting effects. Due to all these mentioned properties, it deserves much more than simply being used as feed. RB forms about 10% of the paddy milled, which corresponds to a very high quantity, since rice is one of the main crops produced worldwide. Reusing such valuable by-products is a key sustainability strategy. RB should either be stabilized or exposed to a process suitable for the end product to which it will be transformed as soon as possible since it deteriorates very rapidly. Although each stabilization method has unique advantages and disadvantages, most of the stabilization methods reported in the literature failed to irreversibly prevent hydrolytic degradation over a long time. Among the stabilization approaches, extrusion, MW, and IR heating seem more promising with regard to industrial scale processing. In addition, steaming and drying (parboiling) is a common and useful process for the RB oil industry. It is critically important to take measures to keep the moisture content of the stabilized RB low throughout storage to prevent reactivation of lipases.

## Data Availability

Not applicable.
